# Search Efficiency Drives Reference Production Across Modalities, But Colour Is Special

**DOI:** 10.1162/OPMI.a.337

**Published:** 2026-02-15

**Authors:** Merrick Giles, Paula Rubio-Fernandez, Francis Mollica

**Affiliations:** Melbourne School of Psychological Sciences, The University of Melbourne, Parkville, Victoria, Australia; Max Planck Institute for Psycholinguistics, Nijmegen, The Netherlands

**Keywords:** reference production, overinformativeness, experimental pragmatics

## Abstract

When speakers refer to objects in the world, they frequently overinform. Contrary to classical theories in linguistics, we hypothesise that overinformativeness is an efficient means of facilitating listener comprehension: speakers use redundancy to provide their listeners with search-efficient perceptual information. In Experiment One (*N* = 72), we operationalise search efficiency as the ease or difficulty of perceptual discrimination. We borrow methods from psychophysics to manipulate discriminability across attributes (material, colour) and across sensory modalities (audition, vision). We show that across both attributes and modalities, speakers overinform more often when the redundant information helps the listener perceptually discriminate the referent, thus aiding listener-search. Contrary to our expectations, we also find that speakers disproportionately overinform with colour information (relative to material). In Experiment Two (*N* = 97), we investigate the disproportionate use of colour directly, addressing explanations that appeal to colour’s perceptual salience by 1) dampening colour’s perceptual distinctiveness, and 2) controlling linguistic complexity both in terms of language production and retrieval effort. Contrary to widespread explanations, these factors cannot explain the disproportionate use of colour: colour is privileged in reference.

## INTRODUCTION

Reference in the physical world is often *overinformative*: speakers say more than required (Pechmann, 1989; Sedivy, 2003). For example, a speaker might say “pass me the blue cup” when there is only one cup on the table, making *blue* redundant. Overinformativeness provides a striking counterexample to classical notions of communicative efficiency (Grice, 1975; Zipf, 1949), which argue that efficient speakers should say as much as necessary, but no more.

Recent theories argue that overinformativeness *is* communicatively efficient if we define the goal of communication in terms of both accuracy and *speed*. As speakers often want listeners to both quickly and accurately identify targets, they may (consciously or not) provide redundant but perceptually useful information to aid the listener’s search for the target (Jara-Ettinger & Rubio-Fernandez, 2022; Rubio-Fernandez, 2019). This *search efficiency* view defines efficiency in terms of the trade-off between the speaker’s production costs and the listener’s search costs. If an expression makes the listener’s search for the referent faster, then the expression may be efficient even if it exceeds strictly necessary informational requirements (Jara-Ettinger & Rubio-Fernandez, 2022; Kursat & Degen, 2021; Rehrig et al., 2021; Rubio-Fernandez, 2019; Tourtouri et al., 2021). To the extent that redundancy facilitates search, it may be worthwhile to suffer the increased production costs (cf. Engelhardt et al., 2011, who argue overinformativeness may not be worth the cost). Concretely, the search efficiency view predicts that the rate of overinformativeness should track with the search efficiency gained by adding a guiding feature to the target’s specification.

The reference production literature provides a wide range of empirical support for the search efficiency view, using manipulations of search efficiency that are directly informed by literature on visual search. In a visual search task, a participant is presented with a scene of objects and is tasked with finding one or more target objects based on a specified criterion. For example, participants might be asked to search for the letter “K” in an array of random letters (Neisser, 1964). Participants then allocate attention to the objects in the array until they find the target. Although there is a longstanding debate as to whether people allocate visual attention serially to each object or allocate attention to some aspects of objects in parallel, both sides agree that there are several privileged visual features that facilitate search for the target (Wolfe & Horowitz, 2004), including colour, orientation, motion, and size (Wolfe & Horowitz, 2017).

**Figure 1.**
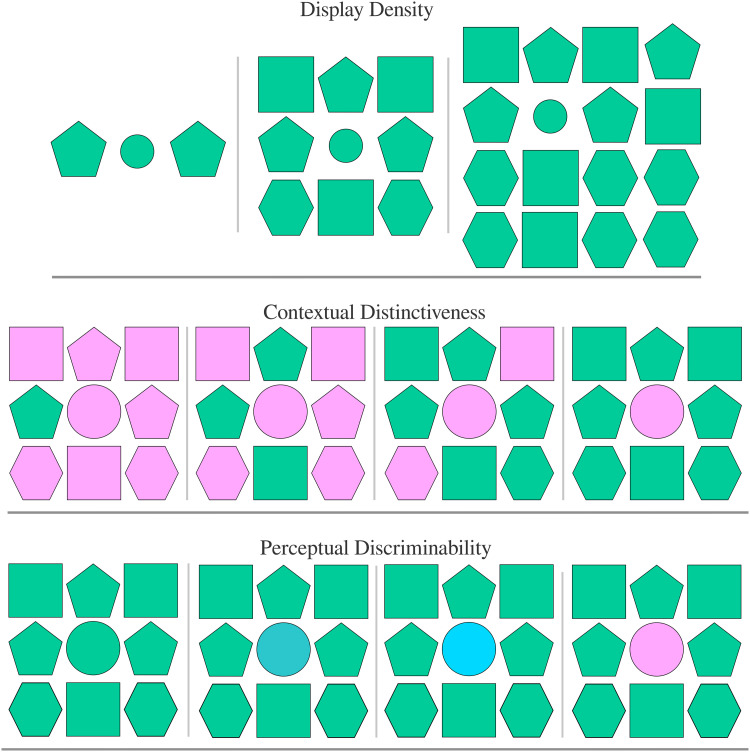
Illustration of the three relevant findings from visual search. In all cases, the target is the circle. Display density, contextual distinctiveness, and perceptual discriminability all increase from left to right. The search efficiency view of reference predicts that rates of overinformativeness using colour/size increase left to right in all cases.

There are three key findings from visual search that are particularly relevant for referential search—each is illustrated in Figure 1. First, all else being equal, when the number of items in a display increases, search time increases due to serial search (Wolfe, 1998). The co-variation of search time with the number of items is known as a *display density effect*. Second, if the target has a privileged visual feature (e.g., colour), pre-attentive filtering can guide visual search and reduce the set of items in the display that need to be serially attended. The impact of this effect depends on the *contextual distinctiveness* of the target. For example, if the target is the only blue item in a nine-item display, search would be extremely fast because attention can be pre-allocated directly and exclusively to the blue item. If half of the items in the display are blue, the visual search time is reduced by about half compared to a serial search of all items. Whereas if all items are blue, pre-allocating attention to blue items has no effect on search times. Thus, search time is an approximately inverse function of contextual distinctiveness (Egeth et al., 1984).

Finally, attribute-guided search is faster when the attribute values of each object are perceptually distant from each other (Duncan & Humphreys, 1989)—i.e., items are *perceptually discriminable*. For example, searching for a blue target amongst a display of jades and purples will be slower than searching for a blue target amongst pinks and yellows due to the greater perceptual distance between blue and yellow/pink than between blue and jade/purple (Wolfe & Horowitz, 2017). In the case of dense displays, these differences dramatically cut visual search times as attention is attracted to large perceptual differences along the privileged visual features—i.e., high contrasts. However, the facilitatory search effects of discriminability are not limited solely to privileged features. Even for non-privileged features, search is slow when the target is perceptually proximate to the surrounding items in the display, and fast when the target is perceptually distant from surrounding items (Alexander & Zelinsky, 2011).

Reference production findings dovetail remarkably well with visual search predictions, best illustrated by experiments manipulating the interaction between display density and the contextual distinctiveness/perceptual discriminability of colour. Overinformative expressions with redundant colour modifiers linearly increase as the density of the display increases (Clarke et al., 2013; Gatt et al., 2017; Rubio-Fernandez, 2019). The increase of overinformativeness in dense displays is particularly pronounced when the target referent’s colour is contextually unique (increases by a factor of seven; Rubio-Fernandez, 2021) and/or highly discriminable (increases by a factor of three to four; Jara-Ettinger & Rubio-Fernandez, 2022; Kursat & Degen, 2021). Whether conscious or not, speakers’ overinformative colour reference in these experiments would provide a listener with facilitatory search-guiding target criteria. Importantly, this facilitative effect has been confirmed with direct referential comprehension measures: contextually distinctive and/or perceptually discriminable information facilitates referential comprehension, whether measured by EEG (Tourtouri et al., 2019; Tourtouri et al., 2021), eye-tracking (Rehrig et al., 2021; Rubio-Fernandez, 2021), or response times (Paraboni et al., 2007; Rubio-Fernandez, 2021).

While common, display density manipulations are not strictly needed for facilitatory overinformativeness. For example, speakers overinform with colour more often in polychrome rather than monochrome displays, irrespective of density, showing a sensitivity to contextual distinctiveness alone (Koolen et al., 2013; Long et al., 2021; Rubio-Fernandez, 2016). In the polychrome case, colour is more contextually distinctive than the bare-noun description, facilitating search. In the monochromatic case, overinformativeness is no longer contextually distinctive—failing to facilitate search, but also more likely to slow search by creating an unresolved ambiguity in the listeners’ search process. This is one of the cases where overinformativeness is inhibitory for listener search (see also Engelhardt et al., 2006; Fukumura & Carminati, 2022). Appropriately, speakers overinform less often when it would be inhibitory, and more often when it is facilitatory. Speakers are remarkably sensitive to whether colour is inhibitory or facilitatory: rates of overinformativeness increase as a function of contextual distinctiveness (Rubio-Fernandez, 2021).

Similarly, manipulations of perceptual discriminability alone can elicit overinformativeness patterns that help listener search. For example, rates of overinformativeness for a given attribute correlate with the perceptual discriminability of the attribute even in sparse displays (Jara-Ettinger & Rubio-Fernandez, 2022; Kursat & Degen, 2021). Speakers overinform with colour (“the blue chair”) more than with material (“the metal chair”), which coincides with colour being more perceptually discriminable than material as measured by participant ratings and visual search times. The same pattern occurs within colour displays—i.e., not just between attributes (Viethen et al., 2017): colours that are highly-discriminable (prototypical colours that are distant from surrounding objects) are referenced in overinformative expressions more often than low-discriminability colours (proximate to surrounding objects, not prototypical). Taken together, speakers’ patterns of overinformativeness closely track with the search efficiency views’ predictions.

Despite such widespread empirical support for the search efficiency view, search efficiency in the reference production literature has only been *manipulated* using colour stimuli: one of the few features privileged in visual search (Wolfe & Horowitz, 2017). While contextual distinctiveness effects depend on privileged attributes such as colour, perceptual discriminability effects do *not* depend on privileged attributes. Yet even with respect to perceptual discriminability, support for the search efficiency view of reference is limited. Prior studies investigating perceptual discriminability only compare rates of overinformativeness between colour vs. material in a cross-attribute comparison. Robust support for search efficiency requires within-attribute manipulation of perceptual discriminability using a non-colour, non-search-privileged attribute. To date, such support is lacking (see Kursat & Degen, 2021). Manipulating perceptual discriminability *within* other absolute^1^ attributes like material poses a methodological challenge: unlike colour, visual attributes like material are inherently limited in their perceptual range. To get around this challenge, we adopt a novel reference task requiring multi-modal search, manipulating the perceptual discriminability of material in the auditory domain. While visually discriminating material constitution is difficult, humans can easily discriminate between a range of materials from their impact sounds alone (Giordano & McAdams, 2006).

If search efficiency is successful as a general theory of reference, manipulations of auditory search efficiency using material attributes should produce precisely the same effects as manipulations of visual search efficiency using colour. In Experiment One, we test this directly. Concretely, we test whether search efficiency, as perceptual discriminability, drives reference for auditory material: will speakers overinform more often with high (rather than low) discriminability material attributes? Moreover, is the influence of search efficiency equal for both visual colour and auditory material?

## EXPERIMENT ONE: DOES SEARCH EFFICIENCY DRIVE OVERINFORMATIVE REFERENCE ACROSS ATTRIBUTES AND MODALITIES?

**Figure 2.**
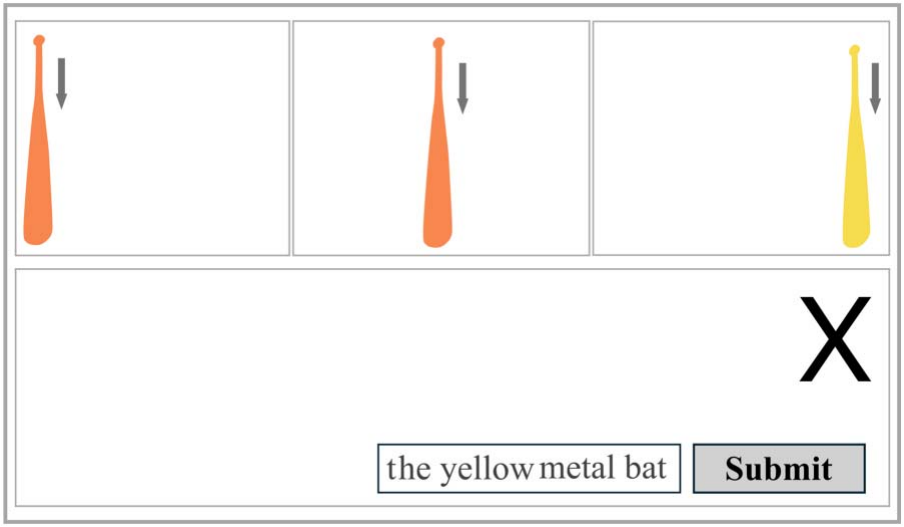
Illustration of participants’ view of a director task trial. Participants observe three bats, all of which fall from the top of the screen to the bottom, where they hit an invisible surface. The moment the bats hit the surface, participants hear the auditory impact sound of wood or metal, of high or low discriminability (depending on the trial condition). In this trial, colour is highly discriminable, and *Sufficient* to establish reference. Note that the bats do *not* remain on the screen after impact. Bats are presented sequentially left-to-right, *then* the *X* appears in the target’s location.

In the first experiment, we introduce a novel variant of the classic director task (Krauss & Glucksberg, 1977): the baseball bat factory. Participants are told they are working as quality control at a baseball bat factory. Their job is to describe the selected, defective bat to a co-worker, using its colour (encoded visually) and/or material (encoded auditorily). On each trial, three bats are animated to fall sequentially from left to right (Figure 2). Combining the material sound with the animation creates the impression of an auditory impact occurring due to the bats falling on a surface. After displaying all bat animations and sounds, the target is demarcated with an *X*. Participants must refer to the target bat. To manipulate search efficiency, we vary both the perceptual discriminability of the colours and materials and the information sufficiency of the attributes.

**Figure 3.**
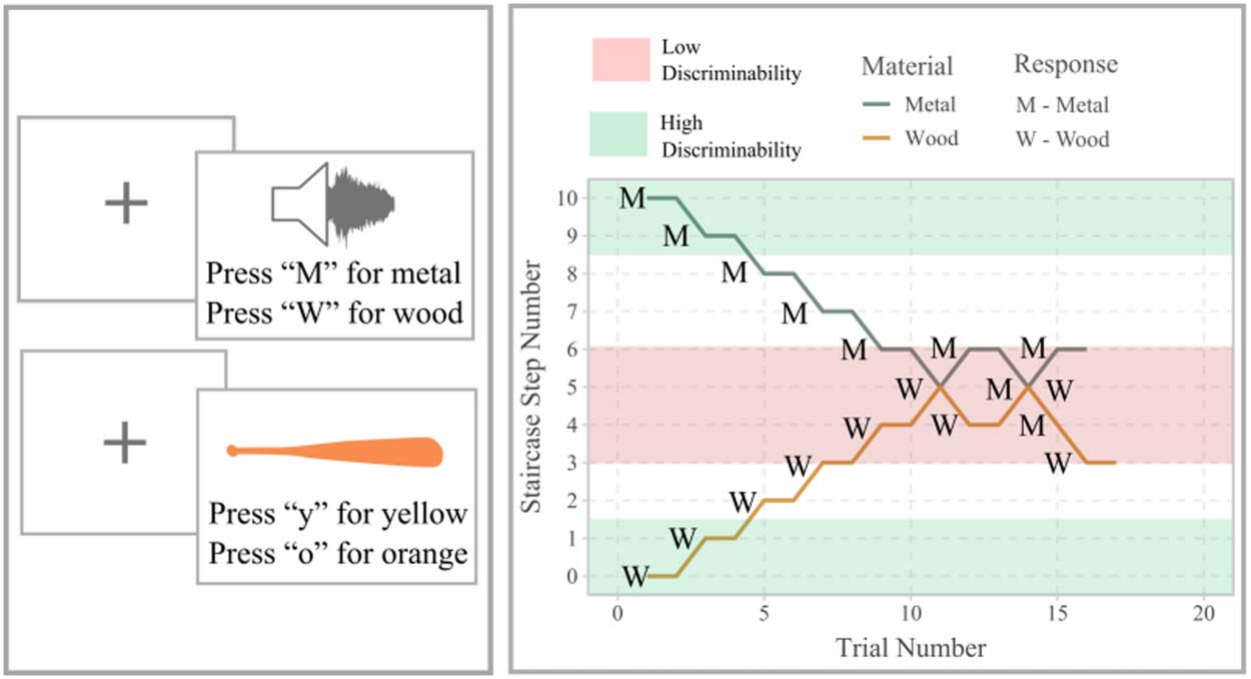
**Left**: Participant view of psychophysical staircases of material (auditory) and colour (visual). Participants classify sounds as wood or metal, and colours as yellow or orange/blue or green. **Right**: example of a single staircase for one (real) participant. With each correct answer, participants move to the other side of the continuum. Materials are difficult to distinguish towards the middle of the continuum (red shading). We identify low discriminability sounds by identifying the location where participants switch from labelling sounds as ‘wood’ to labelling them as ‘metal’ and vice versa.

To ensure that we manipulate perceptual discriminability robustly in light of presentation display differences, perceptual differences, and categorisation differences across individuals, we measure perceptual discriminability using an adaptive staircase method from psychophysics (Leek, 2001; Figure 3). In a psychophysical staircase, participants make categorisation judgements about stimuli to ascertain their categorisation boundary. Based on their performance, we can then select a stimulus that a participant consistently categorises (high discriminability) and the stimuli that a participant most inconsistently categorises (low discriminability) for each dimension. In addition to providing calibrated stimuli, grounding our manipulation of discriminability in participant behaviour allows us to equate discriminability across dimensions, regardless of inherent physical differences between stimulus dimensions. Using the staircase procedure, we reliably derive a set of four stimuli calibrated for each individual participant: high- and low-discriminability colours (visual), and high- and low-discriminability materials (auditory). With these individualised points of high- and low-discriminability colours and materials, we construct bat factory stimuli that manipulate search efficiency.

We manipulate information sufficiency by varying the redundancy of attributes in a trial. An attribute is sufficient if the attribute alone provides enough information to successfully demarcate the target object. For example, consider a scene with one green/metal bat and two blue/metal bats. The colour attribute is sufficient: if a speaker says “the green bat”, the target is successfully demarcated. In the same example, the material attribute is redundant: a speaker need only mention colour, and if they say “the green metal bat”, the expression is an overinformative, material-redundant expression. Analogously, we can have conditions where the material attribute is sufficient, and colour redundant: a scene with one green/metal bat and two green/wooden bats.

We use the interaction between perceptual discriminability (high vs. low) and information sufficiency (sufficient, redundant) to create conditions where redundancy boosts the search efficiency of an expression, and conditions where redundancy does not. When a redundant attribute is high discriminability, overinformativeness boosts search efficiency. This is because the redundant adjective grounds reference in a highly discriminable, search-efficient attribute. When a redundant attribute is low discriminability, however, overinformativeness does not boost search efficiency. A redundant adjective in this case would ground reference in a low-discriminability attribute that does not help search. However, the search efficiency of overinformativeness also depends on the sufficient attribute. If the sufficient attribute is low-discriminability, and the redundant attribute is high-discriminability, there should be a search efficiency pressure for overinformativeness. This is because search with the sufficient attribute is difficult, creating the need for redundancy to guide listener search. If the sufficient attribute is high-discriminability and the redundant attribute is low-discriminability, there is no search efficiency pressure for overinformativeness. In this case, the sufficient attribute is search-efficient as is, and the low-discriminability redundant attribute is not helpful for search. This leads us into three display type conditions, where the search efficiency view makes concrete predictions:

Condition 1: Sufficient-High/Redundant-Low (S-High/R-Low), where the sufficient attribute is of high-discriminability, and the redundant attribute is of low-discriminability. The search efficiency view predicts low rates of overinformativeness here, because a) the sufficient attribute is good for search as is, and b) the redundant attribute does not help search.

Condition 2: Sufficient-Low/Redundant-High (S-Low/R-High), where the sufficient attribute is of low-discriminability, and the redundant attribute is of high-discriminability. The search efficiency view predicts higher rates of overinformativeness here, because a) search using the sufficient attribute is difficult, and b) the redundant attribute helps search.

Condition 3: Baseline, where the sufficient attribute *and* the redundant attribute are both of high discriminability. This condition lets us tease apart reference production strategy at a fine-grained level. If speakers follow a simple strategy of mentioning all high-discriminability attributes of a scene (as argued by Fukumura & Carminati, 2022), overinformativeness should be equal with the S-High/R-Low condition. If speakers use redundancy to help an otherwise difficult search (as argued by Rubio-Fernandez, 2019), overinformativeness will be lower than in the S-High/R-Low condition.

**Figure 4.**
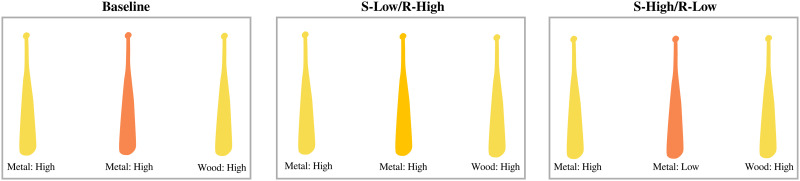
Illustration of the Experimental Conditions. In all three trials in this illustration, the referential target is the bat in the middle. Also in the illustration, *Colour* is the sufficient attribute (presented visually, as shown) while *Material* is the redundant attribute (presented auditorily, but written on the figure for illustration). Note that the low–discriminability colour stimuli vary between participants.

All three display types are counterbalanced as to whether material or colour is the redundant attribute (colour-sufficient/material-redundant, and material-sufficient/colour-redundant). As a result, we can compare S-Low/R-High to the two other conditions for material and colour independently. The three display type conditions are illustrated in Figure 4. We use the colour-sufficient, material-redundant case for illustration.

### Methods

#### Participants.

As we planned to use a complex psychophysics task online, we conducted pilot studies to check that the staircase task was able to determine adequate high- and low-discriminability stimuli for the director task. We expected difficulty running such a task online: we have less control to adapt to differences in people’s perceptual ability, and less control over presentation equipment than in a classic lab study. Fortunately, we were able to find adequate stimuli for ∼75% of pilot participants. Expecting to exclude this approximate proportion of participants from the director task for lack of adequate stimuli, we recruited 120 native English speakers with normal or corrected-to-normal vision, and no colour-vision or hearing impairments through Prolific: we ensured the lack of vision or hearing impairments by using Prolific’s screeners, and double checked by surveying participants at the end of the experiment. We expected a total of ∼80 participants in total. Participants completed the experiment online, on their personal computers. The entire experiment took each participant approximately 40 minutes. Consistent with ethical approval, participants were compensated at a rate of £9.55 per hour.

#### Procedure.

##### Assignment.

To ensure our results generalise across various colours and materials, participants were randomly assigned to one of four conditions, corresponding to a colour-material combination pair. Hard materials (metal, glass) and soft materials (wood, cardboard) were always paired, because people struggle to distinguish metal vs. glass, or wood vs. cardboard, but can easily distinguish between our hard-soft pairings (Traer et al., 2019).

1. wood-glass/blue-green
2. cardboard-metal/blue-green
3. wood-glass/orange-yellow
4. cardboard-metal/orange-yellow

For realism with respect to the baseball bat cover story, materials were always labelled ‘wood’ and ‘metal’ from the participant’s point of view—i.e., glass was labelled as metal, and cardboard labelled as wood. Illustrated in the next section, participants’ perceptual categorisations were not influenced by this deceit, consistent with Traer et al. (2019)’s findings.

##### Adaptive Psychophysical Staircases.

First, participants proceeded through the adaptive perceptual staircases to identify regions of high and low perceptual discriminability for both colour and material. For each attribute, participants completed the two staircases simultaneously—at opposing ends—with interleaved trials. For example, participants began one staircase beginning at wood and ending at metal, and one beginning at metal and ending at wood simultaneously (as labelled in their respective conditions).

Each staircase began with a high-discriminability stimulus. When participants correctly label the material source of the sound, they progress in the staircase (towards the other end of the perceptually smooth continuum). Note that our task is unusual in that there is no inherently correct identification, but a label is considered correct if is it the label associated with the starting stimulus: e.g., if the staircase starts with metal, and a participant identifies a stimulus as metal, this is classified as a correct identification; if they identify a stimulus as wood in the same staircase, this is classified as an incorrect identification.

With two consecutive correct material identifications, participants progressed one step in the staircase. Due to its larger number of steps, the colour staircases were altered to facilitate more rapid completion: with two consecutive correct colour identifications, participants progressed five steps, but only until their first incorrect identification. After their first incorrect identification, they progressed four steps; after their second incorrect identification, two steps; after their third, they progressed through the staircase one step at a time. When participants incorrectly classify a stimulus, this is called a reversal. For example, a reversal occurs when a participant identified the previous step as wood, but then identifies the current step as metal. In the material staircases, incorrect identification was followed by a one-step regression, toward the high-discriminability end of the staircase. In the colour staircases, the aforementioned alteration of larger step jumps was made: on their first incorrect identification, participants regressed four steps; on their second incorrect identification, three steps, and so on. After five reversals, participants were required to correctly label the stimulus once more (likely when the immediately preceding response was incorrect). This ensured that participants were, in fact, willing to classify the low-discriminability stimuli into their corresponding categories.

The stimulus of the final correct identification was defined as the stimulus of low discriminability. A stimulus was defined as high discriminability if it met three conditions: 1) The participant had no reversals in the staircase thus far; 2) The stimulus was “correctly” identified as its source colour/material; and 3) the current step in the continuum was less than or equal to two. If the staircase successfully identified high- and low-discriminability stimuli for both materials and both colours, participants progressed to the reference production director task, where these stimuli were implemented.

##### Reference Production Test: The Director Task.

**Figure 5.**
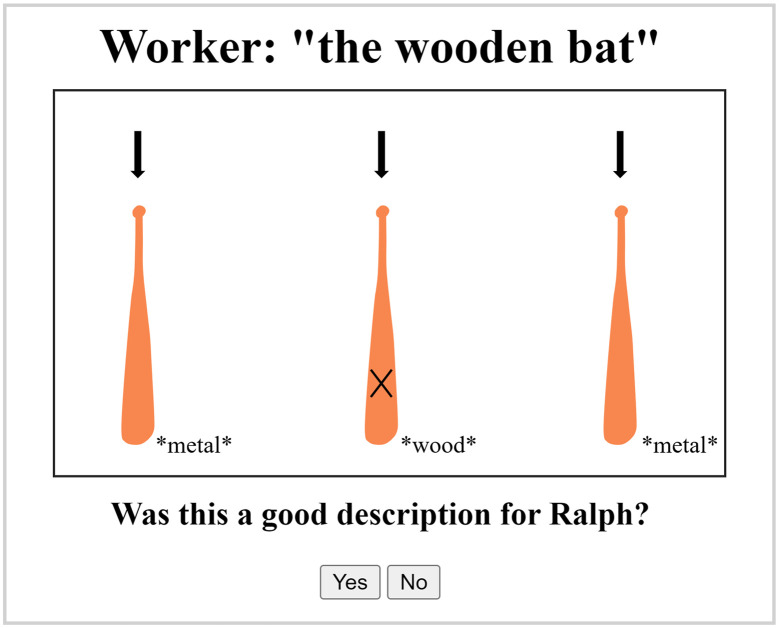
Illustration of the practice phase, undertaken for participants to understand the director task. Participants were asked whether the provided descriptions, such as “the wooden bat” were suitable for the listener (Ralph). Any expression that uniquely demarcated the target (sufficiently or over-informative) using colour and/or material was considered correct. Note that materials in the experiment were presented auditorily, but are written in the figure for illustration.

Following the staircases, participants completed the reference production task. Here, they described baseball bats with an associated colour and material to a hypothetical listener (Krauss & Glucksberg, 1977, see the Director Task). Participants were introduced to an image of their hypothetical listener (named Ralph), and were informed that Ralph was equally adept at finding bats using material or using colour. They were told that they would be describing the bats to Ralph, who was at another station, and that Ralph would be selecting bats to discard based on their description. Before the data-collection phase, participants were tested on their comprehension of the instructions by judging whether the provided descriptions were suitable for finding the target (Figure 5). This comprehension phase consisted of six trials where participants were given a description and watched the baseball bats fall with their associated colour and sound. The target was demarcated with an *X*, and they were asked: “Was this a suitable description for Ralph?” and were required to answer yes or no appropriately (Figure 5). Wrong answers were given feedback with an explanation. We did not require repetition, as we expected participants to rapidly understand the nature of the task with this demonstration. Fillers and critical trials were presented in random order, and we counterbalanced whether colour or material was sufficient or redundant.

In each trial, three bats fell from the top of the screen in a sequence from left to right (Figure 2). Each bat fell for 800 ms before hitting the invisible surface on the bottom of the screen, where they produced the material impact sound (1 s in duration). They remained on the screen (visually) for an additional 1.7 s before disappearing as the following bat began to fall. Once all bats were presented and had disappeared, an *X* appeared at the target’s location, and participants provided their response as a description in the text box.

We employed moderate feedback during the reference-production (director) phase. If participants failed to uniquely demarcate the referent from the surrounding objects, a thumbs-down appeared on the screen. If participants succeeded in unique demarcation, a thumbs up appeared (regardless of whether the expression was overinformative or not). If participants overinformed on filler trials, a character appeared, depicting a shrug. The implementation of the shrug arose during piloting, as we observed that some participants adopted a strategy of systematic overinformativeness on every trial. Adding this graded acceptability to the cover story (some expressions are sometimes better, worse, or in-between) was designed to prompt participants to consider *when* they should apply an overinformative strategy and when they shouldn’t, without ever biasing participants toward overinforming with a particular attribute or display type.

#### Materials.

##### Material Staircase.

To construct materials for the material psychophysical staircases, we synthesised material-derived impact sounds using the generative model built by Traer et al. (2019). Psychophysical staircases require a continuum from one stimulus to the other, such that the stimulus features continuously change. To achieve this, we employed a linear interpolation of 10 equally spaced steps between the modal parameters of sound pairs (amplitude, centre frequency, decay rate). This linear interpolation creates a continuous, perceptually smooth, gradient transition between each material in a pair. The volume of all sounds was normalised to ensure consistency.

To ensure our results generalised across multiple different auditory stimuli, we synthesised two distinct auditory material continuums. As stated in the Procedure section, people cannot distinguish between hard materials such as metal and glass, or between soft materials such as wood and cardboard (Traer et al., 2019). For this reason, our continuums extended across the hard/soft distinctions. One continuum used two synthesised impact sounds of wood and glass, interpolating into a smooth wood-to-glass continuum. The other used cardboard and metal impact sounds, interpolating into a smooth cardboard-to-metal sound continuum. Recall that, for scenario realism, materials were always labelled as “wood” (wood and cardboard) or “metal” (metal and glass) from the participant’s perspective.

##### Colour Staircase.

As with the material staircases, we required a smooth continuum between two colours. The CEILAB space has approximate perceptual uniformity (Brainard, 2003), making it the best space for creating a continuum between two coordinates. We used linear interpolation to create a continuous and perceptually smooth gradient transition between colour coordinates in the CEILAB space (lightness, red-green, yellow-blue).

Like with material, we wanted to ensure our results generalised across multiple different colour stimuli. For this reason, we again created two distinct colour continuums. In the case of colour, it was paramount to generalise our results across warm and cool poles in colour space. English terms support more precise communication for warm rather than cool colours (Gibson et al., 2017; Zaslavsky et al., 2019); the physical spaces between categories are therefore smaller at the warm pole. We therefore created one warm continuum (yellow-to-orange, to which half of the participants would be assigned) and one cool continuum (green-to-blue, to which the other half of the participants would be assigned). In CEILAB space, these coordinates were from blue (73.03, −19.98, 37.84) to green (87.24, −62.94, 41.00), and from yellow (88.29, −0.98, 69.51) to orange (68.55, 40.91, 49.37).

Piloting revealed that participants have highly specific category distinctions for colour, but that these distinctions vary greatly between participants. Consequently, identifying a low-discriminability region of colour space required continuums of a greater number of steps than did material: we employed 50 equally spaced steps for yellow-to-orange, and for blue-to-green. This had little impact from the participants’ point of view, because the participants rapidly jumped through the colour staircases to their personal region of low-discriminability (see Adaptive Psychophysical Staircases in the Procedure section).

##### Bat Factory Trials.

In the reference production task, there were 48 critical trials (24 colour-redundant, 24 material-redundant), which were split into 12 Baseline trials, 12 Sufficient High/Redundant-Low (S-High/R-Low) trials, and 12 Sufficient-Low/Redundant-High (S-Low/R-high) trials. To make it clear that overinformativeness was not necessary, we included an additional 12 filler trials. These were designed so that participants would perceive only one clear difference in either colour or material in a uniform set. For example, stimuli are uniform in material and uniform in colour, except for the target, which differs in colour. Such filler trials make it clear that there are cases where participants do not need not mention both colour and material (overinformative).

### Results

From our initial sample of 120 participants, we removed participants based on a stringent exclusion criterion. Forty-three participants did not have adequate high- and low-discriminability stimuli to complete the director tasks, leaving us with 77 participants. We excluded an additional five participants for failing to sufficiently establish reference (underinformativeness) on >10 trials in the director task (which usually has near perfect accuracy).^2^ In line with prior research, we restricted analyses to trials with identifiable references. The excluded non-identifiable references were approximately equal across colour- and material-redundant trials (for mislabelled attributes, 42% were material-redundant and 58% were colour-redundant), and constituted <5% of the individual participants’ trials. Such inaccuracies were therefore attributed to brief lapses of attention, and only those individual trials were removed (298 trials total). We retained low-discriminability trials with mislabelled attributes, as mislabelling is an inevitable aspect of low-discriminability. There were 325 total mislabelled low-discriminability trials, approximately equal across material-redundant (44%) and colour-redundant (56%), again confirming comparable discriminability for both material and colour stimuli. Finally, one random trial from each participant failed to record due to software error. While the number of participants was slightly less than anticipated (details in Supplementary Materials), another stage of recruitment was not feasible.

The remaining data from critical (non-filler) trials (72 participants, 3481 trials, 2776 critical) were analysed using R (R Core Team, 2023, Version 4.3.2). We employed Bayesian Mixed Effects Logistic Regressions (main effects and by-subject intercepts) using brms (Bürkner, 2017) with 𝒩(0, 2) priors over regression coefficients, chosen using prior predictive checks. Each analysis used dummy-coded contrasts (references: Material-Redundant, S-Low/R-High).

**Figure 6.**
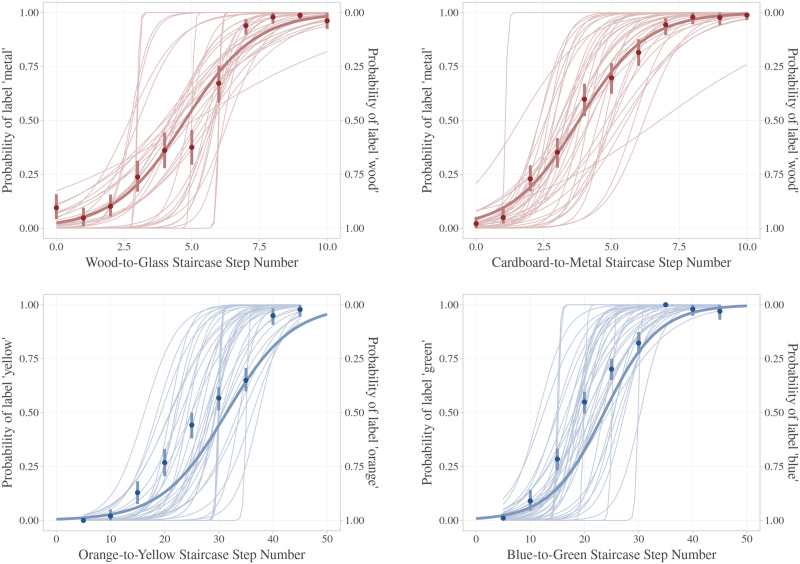
Logistic functions showing discrimination thresholds for both colour and material differences. The probability of discriminating a stimulus as belonging to some category (e.g., ‘metal’) progressively declines as the stimulus characteristics continuously shift from one end of the continuum to the other. This plot confirms high-discriminability as having a high probability of some response (e.g., all participants label the stimulus ’green’ on every trial). We can confirm low-discriminability as some response approximating chance (e.g., the probability of labelling the stimulus ‘green’ or ‘blue’ is 50/50). Operationalising perceptual discriminability in this way, we can manipulate discriminability as an underlying source of both colour (visual) and material (auditory) comparatively. The dark, thick line shows the average Logistic function across all participants. The light, thin lines show logistic functions of individual participants. Points show the average probability of the label for that single stimulus as a point in the staircase, with line-ranges showing bootstrapped 95% confidence intervals. Note that for colour, individual points are binned per five trials for reliable confidence intervals.

Before analysing the director task, it’s important to demonstrate that our manipulation of perceptual discriminability via the staircases was successful. Figure 6 shows the categorisation curves for each continuum. For most participants, the curve reflects a smooth, continuous shift between certain categorisation of one end (e.g., wood) and certain categorisation of the other end (e.g., metal) of the continuum, which allowed us to extract clear “certain” and “uncertain” stimuli as high- and low-discriminability, respectively. For a few participants, the curve is nearly vertical, suggesting highly precise discrimination boundaries. However, even these participants have at least one stimulus where they do not perform above chance in discrimination accuracy.

To recap, the Search Efficiency view predicts that 1) rates of overinformative reference will be higher for S-Low/R-High displays than S-High/R-Low displays and Baseline displays. If discriminative ease or difficulty is the key driver of Search Efficiency, rates of overinformativeness between attributes (Colour-Redundant vs. Material-Redundant) should be no different.

**Figure 7.**
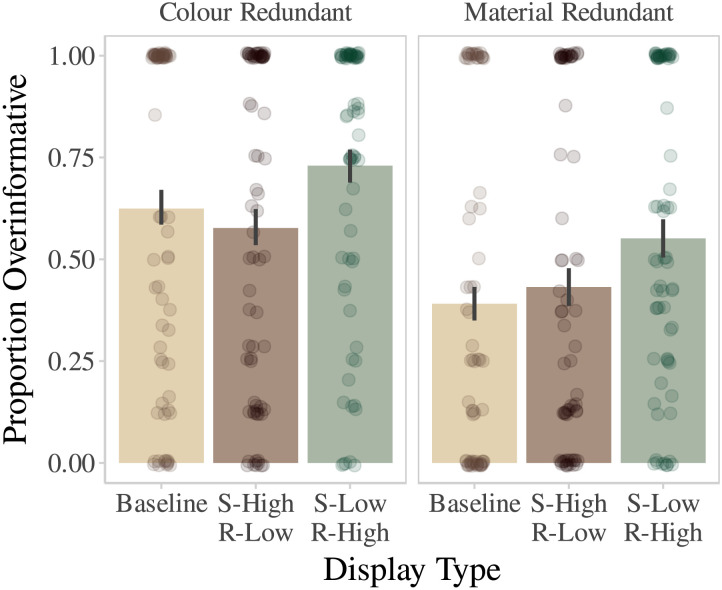
Dots represent individual participant means. Lines represent bootstrapped 95% bootstrapped confidence intervals.

As can be seen in Figure 7, participants were more likely to overinform in the S-Low/R-High display type compared to baseline (both attributes high-discriminability) and S-High/R-Low. Comparison of the S-High/R-Low and Baseline display types to the reference level of S-Low/R-high shows that participants overinformed more often when doing so was required to ground reference in a high-discriminability attribute. The results for display types precisely follow the predictions of the Search Efficiency view.

Rates of overinformativeness were higher when colour, rather than material, was the redundant attribute (*β* = −1.43, 95% CI = (−1.65 – −1.20). As the discriminability of redundant colour and material information was controlled using the staircase, search efficiency—formalised as discriminative ease or difficulty—cannot account for this result. The complete statistical results are presented in Table 1.

**Table 1.** Regression results.

Effect	*β*	SE	95% CI	
			LL	UL
Intercept	−2.50	0.41	−3.34	−1.70
Material Redundant	−1.43	0.11	−1.65	−1.20
Baseline	−0.94	0.13	−1.20	−0.68
S-High/R-Low	−1.09	0.13	−1.35	−0.83

Reference levels: Colour Redundant, S-Low/R-High; SE = Standard Error; 95% CI = Bayesian 95% Credible Intervals. Assumptions of Markov Chain convergence were met with $\hat{R}$ ≈ 1.

Comparing the reported model to a model with an interaction between Redundant Attribute and Display Type, we found no evidence for an interaction between Display Type and Attribute (the model including the interaction was inferior, if only marginally, *BF* = 0.98 to the model without interation: see Supplementary Materials), suggesting the manipulation of Perceptual Discriminability via Display Type extended across both colour and material attributes. The drivers of Search Efficiency extend to non-colour attributes, and even to non-visual modalities.

### Discussion

The key comparison in extending the search efficiency view was the comparison between S-High/R-Low and S-Low/R-High. The search efficiency predicts higher rates of overinformativeness in the S-Low/R-High condition, because search using the sufficient attribute is difficult, and the redundant attribute helps search. In the S-High/R-Low case, the sufficient attribute is good for search as is, and the redundant attribute does not help. The results comparing the two conditions precisely supported the predictions of the search efficiency view: speakers overinformed more often in the S-Low/R-High condition. Moreover, this support extended across both colour (visual) and material (auditory) independently. Across these completely different attributes and modalities, the underlying manipulation of perceptual discriminability showed that rates of overinformativeness followed patterns most useful for efficient listener search. Speakers overinformed most often in S-Low/R-high displays, where overinformativeness was necessary to ground reference in a high-discriminability, search-efficient attribute. In S-High/R-Low displays, the sufficient attribute was already high-discriminability and search-efficient; overinformativeness in these cases would not ground reference in a search-efficient attribute. As predicted by search efficiency, speakers overinformed less often in these cases.

The results also showed that rates of overinformativeness were higher in the S-Low/R-High condition than the Baseline condition (where both sufficient and redundant attributes were high-discriminability). This comparison lets us tease apart reference-production patterns at a fine-grained level. If S-Low/R-High and Baseline were equal, it would suggest that speakers are following a general strategy of mentioning all high-discriminability attributes in a scene, largely ignoring whether attributes are sufficient or redundant. The results provide evidence against this suggestion: the significant difference between the conditions demonstrates that speakers are sensitive to the interaction between search-difficulty and high-discriminability; they are not simply mentioning all high discriminability attributes (baseline), but are mentioning high discriminability attributes in response to a low discriminability sufficient attribute.

While the effect sizes of our perceptual discriminability manipulations in the present study are smaller than those observed in previous studies (e.g., Rubio-Fernandez, 2021), this is very likely due to the influence of *consistency* (Tarenskeen et al., 2015): Within-subject manipulations in reference-production experiments have much smaller effect sizes than between-subject manipulations—likely due to the adoption of a single strategy that persists across experimental conditions. We anticipate that replications of the present experiment with between-subject manipulations will elicit far greater effect sizes; however, this is beyond the scope of the present study.

Despite our search efficiency manipulations, the findings also showed that speakers have higher base rates of overinformativeness with redundant *colour* attributes than with redundant *material* attributes. Of course, material was presented in the auditory modality, and colour in the visual modality. The disproportionate use of colour may therefore reflect a preference for mentioning visual attributes over auditory ones. It is worth noting that such a disproportionate use of visual over auditory attributes is consistent with search efficiency in two potential ways. First, auditory attributes are weighted less than visual attributes in cue integration (Battaglia et al., 2003; Bejjanki et al., 2011), meaning that visual properties are interpreted as inherent features of an object, which speeds up guided search; not so for auditory properties. If visual attributes, but not auditory attributes, speed up search due to cue-integration, the disproportionate use of visual attributes is consistent with the search efficiency view. Second, in general contexts, verifying an attribute by visual inspection is likely faster than verifying through auditory impact sound. A listener can easily verify the colour of an object with a single glance, while verifying material through impact sound requires picking up an object and dropping it. While both aspects of our stimuli were equally verifiable for the speaker, we cannot be certain what inferences our participant speakers made about the listener.

Beyond our particular modality differences, the disproportionate use of colour is not unique to the present study; the finding accords with decades of experiments comparing rates of colour overinformativeness with other attributes, including material (Kursat & Degen, 2021; Sedivy, 2004), pattern (Tarenskeen et al., 2015), and size (Degen et al., 2020; Pechmann, 1989; Rubio-Fernandez, 2019; Sedivy, 2003; Westerbeek et al., 2015). In all tested cases, rates of colour overinformativeness are higher relative to other attributes. Accordingly, we find it unlikely that modality alone drives the disproportionate use of colour in the present experiment; even in prior experiments with visual-only attributes, the disproportionate use of colour remains. Nevertheless, the results of Experiment One suggest that perceptual discriminability does not drive colours’ disproportionate use either. In Experiment Two, we therefore seek to test alternative explanations.

The most widely accepted explanation of colours’ disproportionate use in reference appeals to the notion of *salience*: an overloaded term in cognitive science taken to mean visual conspicuity (Tatler et al., 2011), memory activation (Ariel, 2014), and general surprise (Itti & Baldi, 2009). Most commonly, salience acts as a circular reification of a behavioural observation. In the overinformativeness literature, appeals to salience are most consistent with the latter, although they intend visual conspicuity: colour is said to be an inherently salient attribute and speakers are prone to mentioning salient attributes, commonly overinforming to do so (Arts et al., 2011; Degen et al., 2020; Koolen et al., 2013; Rubio-Fernandez, 2016; van Gompel et al., 2019). For the sake of clear formalisation, we will adopt the visual conspicuity definition of salience, which will allow us to ground salience in terms of search efficiency.

In terms of visual conspicuity, salience arises from the bottom-up (pre-)attentional mechanisms for select visual attributes: colour, orientation, size, and motion (Wolfe & Horowitz, 2017). For these attributes, pre-attentive maps are overlaid on the visual scene, and local contrast operations are performed such that attention is allocated to the sharpest contrast for a given attribute in a winner-take-all manner (Koch & Ullman, 1987). Crucially, these attributes only guide attention if they are highly *contrastive* in the scene—i.e., contextually distinct, and perceptually distant to surrounding objects. As discussed earlier, search time inversely scales with the degree of contextual distinctiveness along privileged attributes (Wolfe & Horowitz, 2017), and thus, rates of overinformativeness scale with contextual distinctiveness (Rubio-Fernandez, 2021). As our study and previous studies have frequently pitted colour—a privileged feature for guiding attention—against non-privileged attributes like material, the high rates of colour over-modification may be due to an additive contextual distinctiveness effect on visual search.

To remedy the confound of attentional privileging, we will investigate whether a different but equally privileged attention-guiding attribute—orientation—produces equally high rates of overinformativeness.

## EXPERIMENT TWO: IS DISPROPORTIONATE COLOUR MODIFICATION DRIVEN BY ATTENTIONAL GUIDANCE, PERCEPTUAL DISCRIMINABILITY, AND/OR CONTEXTUAL DISTINCTIVENESS?

**Figure 8.**
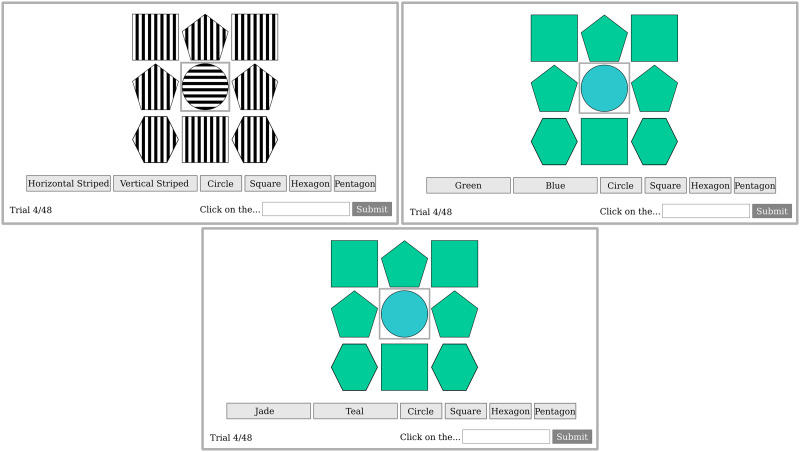
Illustration of Experiment 2 stimuli and conditions. In all illustrated cases, the ***circle*** is the target. The figure presents the three between-subject conditions: Orientation (top left), Colour–Redundant High Frequency (top right), Colour–Redundant Low Frequency (bottom).

In Experiment Two, we implement a classic director task (Krauss & Glucksberg, 1977), where stimuli are presented solely in the visual modality (Figure 8). Participants are presented with a visual array of shapes where one of the shapes is demarcated as the target. Their task is to describe the target for a listener who will see the same display and whose goal is to locate the target. To describe the target, participants can use either adjective-noun combinations or nouns alone. In critical trials, adjective-noun combinations are overinformative; the noun alone demarcates the target.

Across participants, the shapes vary in either colour (teal/jade) or orientation (vertical/horizontal). We confirmed that both colour and orientation had high perceptual discriminability by confirming high labelling accuracy (≥99% accuracy for both colour and orientation). We use perceptually proximate colours (Figure 8) to remove colours’ contextual distinctiveness and perceptual distance. Consequently, we remove colour’s capacity to guide attention, effectively removing its visual conspicuity and therefore its salience. We then compare colour to a contextually distinctive, perceptually distant, attention-guiding alternative: orientation. Unlike prior comparisons between colour and alternatives like pattern (e.g., Tarenskeen et al., 2015) or material (e.g., Jara-Ettinger & Rubio-Fernandez, 2022; Kursat & Degen, 2021; Sedivy, 2004), orientation is a privileged visual feature capable of producing salience effects. And unlike size (another such privileged feature in visual attention), it corresponds to an absolute semantic dimension.

In previous research on overinformativeness, the compared redundant attributes (e.g., colour vs. size) always directly correspond to common, concise adjectives (e.g., ‘blue’, ‘green’, ‘big’, ‘small’). From a search efficiency perspective, it is assumed that the cost of producing the redundant adjective trades off with search-gains in comprehension (Rubio-Fernandez, 2019). Comparing orientation and colour creates a potential confound here, because production costs for orientation (‘the vertically-striped circle’) are likely much higher than for colour (‘the blue circle’). It is therefore imperative that we control production costs so that any observed differences in overinformativeness between colour and orientation can be attributed to perceptual factors. We control for two potential differences in production costs between orientation and colour: 1) word-lengths and 2) word-frequency.

With respect to word-lengths, prior research indicates that speakers aim to minimise production effort (Gibson et al., 2019). This could confound results as colour terms (e.g., blue) are shorter than orientation terms (e.g., vertical) and therefore require less manual effort to produce (e.g., to type on a keyboard). We control for this difference in production effort by using a response selection task where participants click buttons to construct their expressions (Figure 8). In both colour- and orientation-redundant conditions, overinformative expressions require two clicks (adjective, noun).^3^

The second potential production confound is word-frequency. Words that frequently occur in speech and text might be used more readily based on ease of lexical retrieval (Ferreira & Dell, 2000), resulting in more overinformativeness with highly frequent terms (Kursat & Degen, 2021, highlight this possibility). Colour’s disproportionate use might thus be confounded by word-frequency differences. The button-clicking task removes much of this confound, as production no longer requires retrieval. Still, we incorporate additional control: since colour terms ‘green’ and ‘blue’ occur more frequently than orientation terms ‘vertical’ and ‘horizontal’, we include a third condition with low-frequency colour synonyms ‘teal’ and ‘jade’ (Figure 8). If our results are attributable to differences in word-frequency, rather than our perceptual manipulations, then the low-frequency colour condition should have rates of overinformativeness that are at least as low as the orientation condition.

**Figure 9.**
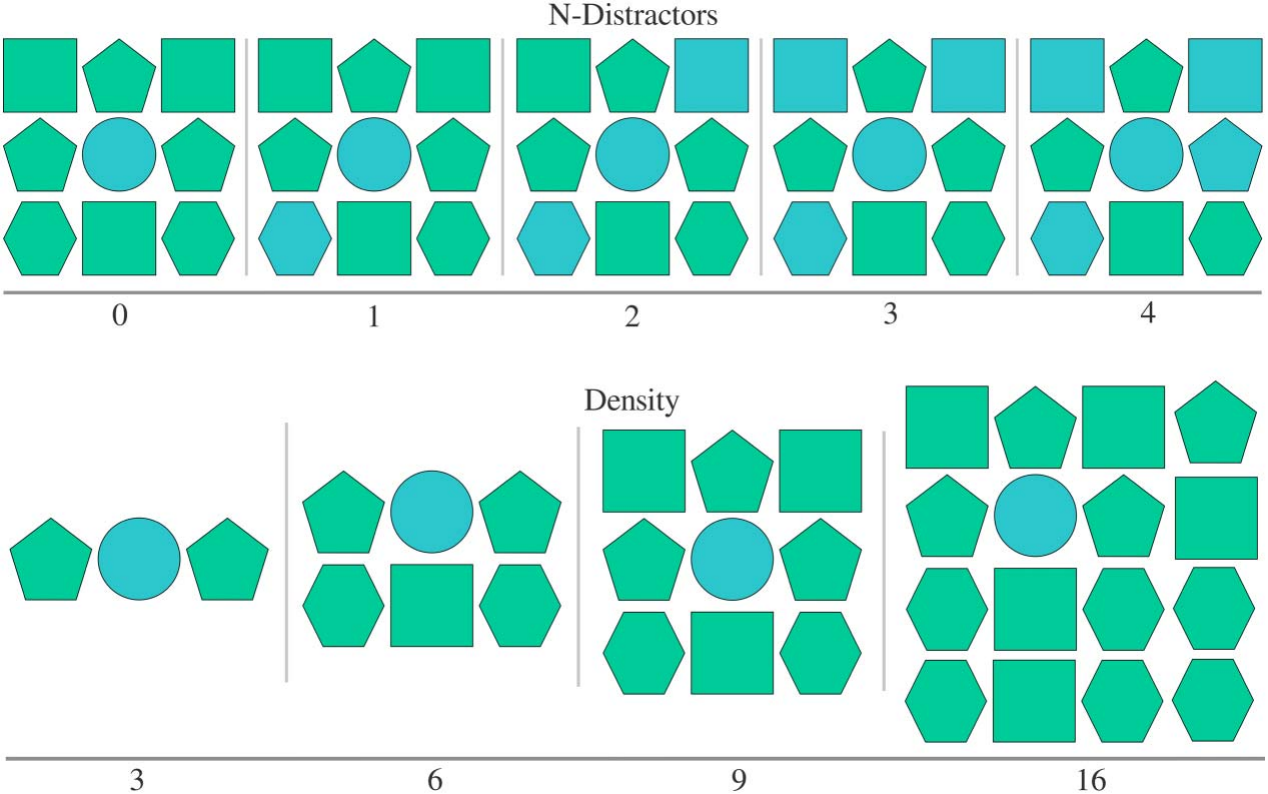
Illustration of Experiment 2 within-subject display types. We use colour (not orientation) for illustration. In all illustrated cases, the ***circle*** is the target. Top illustrates the varying contextual distinctiveness through the number of ‘distractors’ (number of shapes matching the target in the redundant attribute; 0, 1, 2, 3, 4). Bottom shows varying display density as set-sizes (3, 6, 9, 16).

Finally, we wanted to ensure our results comparing colour- and orientation-redundant attributes were robust across display density (Rubio-Fernandez, 2019) and degree of contextual distinctiveness (Figure 1; Rubio-Fernandez, 2021). Display density corresponds to the number of items in the display (three, six, nine, sixteen). The degree of contextual distinctiveness corresponds to the number of items matching the colour/orientation of the target (zero to four). We varied both display density and degree of contextual distinctiveness within-participants (Figure 9) across each of the three between-participant conditions: 1) orientation–redundant, 2) colour–redundant low-frequency, or 3) colour–redundant high-frequency (Figure 8).

To recapitulate the predictions of the search efficiency account, rates of overinformativeness should scale with gains in search efficiency irrespective of attribute. If contextual distinctiveness drives the disproportionate use of colour terms in the previous literature, we would expect orientation and colour to have similar rates of overinformativeness in our experiment, where colour discriminability is controlled. If we find that colour is disproportionately used, it would suggest that something about colour is special beyond search efficiency considerations.

### Methods

#### Participants.

In the absence of complex psychophysical staircases, we expected a far lower proportion of exclusions (both participant and trial) for Experiment Two. Following a power analysis (details in Supplementary Materials) for the smallest effect of interest, we recruited 60 native English speakers (20 per condition) with normal or corrected-to-normal vision through Prolific. Consistent with ethical approval, participants were compensated at a rate of £9.55 per hour.

#### Materials.

To create the colour stimuli, we derived proximate coordinates from CEILAB space extending across the green-blue category boundary (blue: 73, −36.31, −13.94, green: 73, −54.46, 13.65: Figure 8). To prevent any potential contrastive differences that could arise due to differences in luminance, we equalised lightness between coordinates.

To establish attentional–guidance for orientation, the target stimuli were juxtaposed to a *uniform* background—i.e., the stripes of the non-target referent must exactly align on the *x*- or *y*-axis with equal scale. Having established attribute features of colour and orientation, we applied these features to simple nouns: a set of shapes equal in width and height: circles, squares, pentagons, and hexagons (Figure 8). Shapes were chosen for their neutrality, as we aimed to avoid noun-type influencing production patterns, as different object types are known to be associated with varying proportions of colour overinformativeness (Long & Rubio-Fernandez, 2024; Rubio-Fernandez, 2016).

Participants proceeded through 48 total trials (34 critical, 14 filler). Critical trials were constructed by randomly selecting a unique shape for the target (Circle, Square, Pentagon, Hexagon) and randomly selecting the surrounding shapes from the remaining set of possible shapes (excluding the target). Thus, all critical trials required the mention of shape alone (colour or orientation was always redundant). Filler trials were those that required both an adjectival attribute (colour, orientation) and a noun (shape) to establish reference. We implemented these trials to prevent participants from noticing a clear pattern: no critical trial ever required adjectival modification. Assuming we did not include such critical trials, participants would likely notice that only the shape is required on every trial without exception, and default to mentioning shape only.

Trials were further broken down into set size and number of distractors: Set Size 3 appeared with two *N*-distractor conditions (0, 1), while Set Sizes 6, 9, and 16 appeared with five *N*-distractor conditions (0, 1, 2, 3, 4). Participants completed every combination of set size and *N*-distractors twice. We fully randomised the order of Set-Size/*N*-distractor presentation, the positions of target and surrounding objects, and the within-attribute sufficiency type (horizontal vs. vertical / green vs. blue / jade vs. teal).

#### Procedure.

Participants were randomly assigned to one of three conditions: Orientation-Redundant, Colour-Redundant High Frequency (HF), or Colour-Redundant Low Frequency (LF). Participants were informed that they were assigned the role of speaker. On each trial, their task was to construct an expression that gets another participant, the listener, to click on a target object in the same (but shuffled) display. They were informed that the target would *not* be pointed out to the listener. The listener would only be able to use the description placed in the text box. After a comprehension check, participants proceeded through 48 trials (34 critical, 14 filler) whereby they referred to a displayed target surrounded by a grey border (Figure 8). The entire experiment took participants approximately 10 minutes.

Participants constructed expressions by clicking buttons corresponding to attributes (Horizontal Striped, Vertical Striped / Green, Blue / Teal, Jade) and nouns (Circle, Square, Pentagon, Hexagon). When participants pressed a button, the words in the button appeared in the text box. We included the prompt “Click on the …” in the button-click conditions to create more naturalistic expressions. Once satisfied with their expressions, participants clicked *Submit* to move to the next trial. If participants attempted adjective-only responses, a message appeared on the screen informing them that they had to include a noun in their responses. Therefore, if participants chose to mention an adjectival attribute, they were always doing so at the expense of overinforming (they could not simply click “green”). Objects were static, remaining on the screen until participants clicked submit and moved to the following trial (Figure 8).

### Results

From our sample of 60 participants, none met the pre-registered exclusion criteria. We removed individual trials that failed to establish correct reference—i.e., participants mislabelled the target noun. This resulted in the removal of 44 trials: a qualitative check revealed that this was commonly due to participants labelling hexagons as pentagons or vice versa.^4^

The remaining data consisted of 1994 critical trials. Data was visualised and analysed using R (R Core Team, 2023) in the Tidyverse (Wickham et al., 2019). We employed Bayesian logistic regressions (intercept and main effects) using brms (Bürkner, 2017) with 𝒩(0, 2) priors over regression coefficients, chosen using prior-predictive checks. The predictor of Attribute (orientation, colour) was coded using sum contrasts.

As anticipated, rates of overinformativeness across Set-Size and *N*-distractors appeared stable, with no notable effect (see Supplementary Materials). It is therefore unlikely that any effect observed is being driven by these conditions, though note that we did not have the statistical power to assess precise differences statistically.

**Figure 10.**
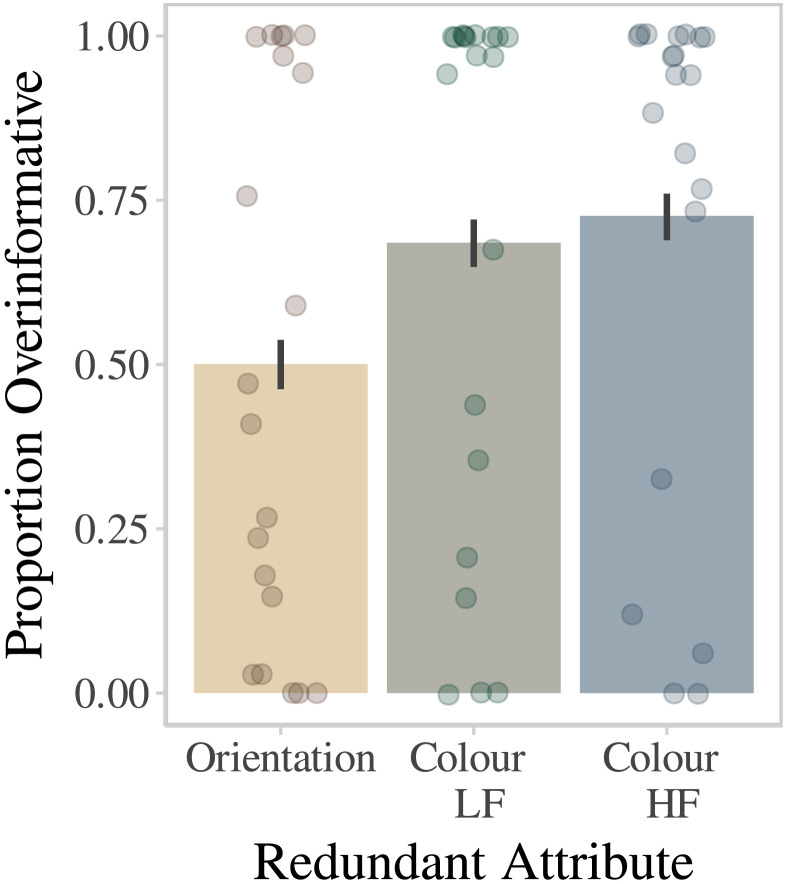
Dots represent individual participant means. Lines represent bootstrapped 95% confidence intervals. LF = Low Frequency; HF = High Frequency.

As shown in Figure 10, participants were far more likely to overinform in both High Frequency and Low Frequency Colour redundant conditions than in the Orientation redundant condition. The difference in the rate of overinformativeness between the colour conditions was negligible. Estimated parameters with credible intervals are presented in Table 2.

**Table 2.** Regression results.

Effect	*β*	SE	95% CI	
			LL	UL
Intercept	0.97	0.09	0.80	1.14
LF Colour Terms Redundant	−0.20	0.12	−0.44	0.03
Orientation Redundant	−0.97	0.12	−1.20	−0.75

Reference level: High Frequency Colour Terms; SE = Standard Error; 95% CI = Bayesian 95% Credible Intervals.

### Discussion

The findings of Experiment Two show that rates of overinformative reference were far higher using redundant colour attributes than redundant orientation attributes. This was the case even despite equalising production effort and word frequency across conditions, and reducing the visual contrast of colour. We can therefore conclude that search efficiency—operationalised as contextual distinctiveness along privileged features (i.e., salience)—cannot completely explain the disproportionate use of colour in overinformative reference.

One potential confound concerns residual linguistic complexity. Despite our controls of production effort, linguistic complexity might still be said to explain the results due to participants’ reading difficulty. The colour terms were shorter than the orientation terms, and therefore may have been easier to read and process. Although we cannot definitively rule this out, we find it unlikely that this drives our results. Across all trials, the available adjective buttons were placed on the screen in precisely the same location. It is therefore likely that participants quickly habituate to the location of the two possible adjective buttons to construct their expressions, making the differences between the conditions negligible after just a few trials. Moreover, the cost of producing orientation adjectives was much higher in the free response version of the experiment (reported in Supplementary Materials), where participants were required to type out their expression. If production effort were a strong factor, we would expect a strong increase in rates of orientation overinformativeness in the button-press version relative to the free response version. Yet, proportions of overinformativeness for orientation between button-press and free-response conditions were almost identical.

## GENERAL DISCUSSION

In Experiment One, we showed that the production patterns of speakers were sensitive to perceptual discriminability, with speakers tailoring their expressions in precisely the ways predicted by the search efficiency view. When the perceptual discriminability of the sufficiently demarcating attribute is low, and the redundant high (S-Low/R-High), we see an increase in the use of redundant but high-discriminability attributes. In these cases, the redundant attributes help the listeners’ search by grounding an otherwise difficult search process in an easy-to-distinguish (albeit redundant) attribute. When the perceptual discriminability of the sufficiently demarcating attribute is high, and the redundant low (S-High/R-Low), we see the reverse pattern; rates of overinformativeness are low. In these cases, the redundant attributes do not help the listeners search (being of low discriminability), and the listeners’ search is grounded in an easy-to-distinguish attribute using a sufficiently informative expression. As predicted by the search efficiency view, rates of overinformativeness in these conditions decline.

Prior work has long supported the search efficiency view using colour stimuli (e.g., Long et al., 2021; Rubio-Fernandez, 2016, 2021). The present findings generalise these results beyond colour: perceptual discriminability drives reference across attributes, across modalities, and even to cases of reference from working memory. While we did not investigate listener search directly using comprehension measures, there are two ways our perceptual discriminability manipulations may implicate listener search: via *perceptual distance*, and/or via *classification difficulty*.

With regards to *perceptual distance*, high-discriminability attributes were perceptually distant from surrounding objects in the display, while low-discriminability attributes were far more perceptually proximate. Large perceptual distances are known to make search easier, and small perceptual distances make search harder (Alexander & Zelinsky, 2011; Wolfe & Horowitz, 2017); consequently, prior work shows that speakers overinform more often with colour when the colour of the target is at a greater perceptual distance from surrounding objects (Rubio-Fernandez, 2019; Viethen et al., 2017). With regards to *classification difficulty*, high-discriminability attributes were prototypical and easy to classify, while low-discriminability attributes were at the threshold between two categorical classifications (e.g., at the threshold between yellow vs. orange), making them difficult to classify. If the attribute is difficult to classify, search is slowed. Likewise, prototypical easy-to-classify attributes are search-efficient. Slow search with classification difficulty has been demonstrated in the reference production literature, and has been used to explain low rates of material overinformativeness (Jara-Ettinger & Rubio-Fernandez, 2022; Kursat & Degen, 2021). Classification difficulty might also explain the low rates of overinformativeness with *size* attributes (Degen et al., 2020; Pechmann, 1989; Rubio-Fernandez, 2019; Sedivy, 2003). Based on the present findings, we cannot ascertain whether perceptual distance or classification difficulty was the most pertinent driver of reference production, and we leave space for future work to disentangle these possibilities.

Another finding of Experiment One was that speakers overinformed with colour far more than with material. An obvious culprit to blame would be modality. While we controlled the discriminability of our stimuli, auditory properties are often less reliable and, thus, weighted less than visual properties in cue integration (Battaglia et al., 2003; Bejjanki et al., 2011). If this expectation of unreliability was carried into our experiment, the disproportionate colour use may closely track with search efficiency. That said, disproportionate colour use has been robustly demonstrated against other visually presented attributes (Arts et al., 2011; Degen et al., 2020; Koolen et al., 2013; Rubio-Fernandez, 2016; van Gompel et al., 2019). These studies appeal to the notion of salience, i.e., visual perspicuity, to explain their results: speakers are prone to mentioning salient attributes, and colour is a highly salient attribute.

In Experiment Two, we define salience in terms of visual search mechanisms (Wolfe & Horowitz, 2017). In visual search, salience arises from pre-attentive filtering directed by perceptual contrasts (perceptual distance and contextual distinctiveness) of privileged attributes such as colour and orientation. We therefore dampened the perceptual distance and contextual distinctiveness of colour and compared rates of overinformativeness between colour displays and contextually distinct, perceptually distant orientation displays. Nevertheless, overinformative reference to colour remained disproportionate, suggesting the widespread ‘salience’ explanation cannot explain the disproportionate use of colour in reference.

Naturally, this leaves open the question of why colour is so ubiquitous in establishing reference. We cannot determine the answer to this question in the present study, but we offer speculations that appeal to speakers’ goals and their prior experience of producing referring expressions.

One possibility is that the disproportionate use of colour falls out of domain-general mechanisms for strategy selection (Lieder & Griffiths, 2017). Through successful and unsuccessful attempts at coordinating reference, speakers learn to predict the speed and accuracy of referential strategies and select strategies that optimise that trade-off. Whether explicitly devising and selecting strategies or implicitly constructing referential habits from their experience (Ferreira, 2019; Rubio-Fernandez, 2021), speakers may learn that colour often leads to successful coordination and more readily deploy that strategy even when its search efficiency in the current display is muted.

Of course, the strategy selection account rests on the assumption that colour has performed better than alternatives in prior search-based contexts. There is some justification for this assumption. Colour categories are highly optimised for *perceptual* communication, maximising the perceptual distance between and minimising the perceptual distance within basic colour categories (Abbott et al., 2016; Regier et al., 2007). In other words, colour space is partitioned to maximise the discriminative capabilities of human vision, making colour categories remarkably search-efficient. While functional optimality of this nature may be a general principle of communication across varied semantic domains (e.g., Kemp & Regier, 2012; Rosch, 1978), the demarcations for material categories such as ‘wood’ and ‘metal’ may be suboptimal with respect to *perception*. Although colour terms are optimised for perceptual demarcation, there is no evidence that this is the case for material distinctions: material might rather be optimised for construction needs, for example. The notion that materials are sub-optimal for perceptual search (unlike colour) is supported by the comparative superiority of colour over material, evidenced by visual search times using real-world objects (Jara-Ettinger & Rubio-Fernandez, 2022; Kursat & Degen, 2021). While orientation shares the superior search efficiency of colour in artificial experimental displays, this might not be true of real-world objects in the environments people generally inhabit. One prediction of this explanation is that an analysis of the naturalistic scenes speakers inhabit will find that colour is a highly search-efficient attribute across contexts. We leave this for future work.

An alternative possibility is that speakers predict that colour reference is a useful strategy by virtue of their communicative goal. Attention is readily deployed to attributes that are goal-relevant in the context despite attentional capture from bottom-up perceptual processing (Henderson et al., 2009). It’s possible that if listeners judge colour as likely relevant to the speaker’s goal, colour reference could be search-efficient regardless of bottom-up attentional mechanisms. Further, if listeners’ search is guided by goal-relevant attributes, then an efficient production strategy is to mention attributes that are the most goal-relevant. There is some indirect support that speakers mention colour when it is more goal-relevant: colour use scales with the goal-relevance of colour variation across environments. In cultures where colour variation is relevant to speakers’ goals, the degree of spontaneous colour use is far higher. This is commonly the case for industrial cultures with high colour variance within object-classes (e.g., the same class ‘shirt’ can take on many colours; Gibson et al., 2017), but likely extends even to non-industrial environments when colour has particular significance to speakers’ goals (Wnuk et al., 2022). Moreover, even within a language community, colour use scales with the goal-relevance of colour. For example, English speakers modify expressions with colour very often for clothes, but far less often for appliances such as vacuums (Long & Rubio-Fernandez, 2024). While this is currently interpreted with respect to listener expectations of informativeness, these expectations likely arise from goal-relevance: colour is more relevant to speakers’ goals involving clothing than it is for their goals involving appliances. The same could occur between attributes: if colour is judged based on prior experience to be more goal-relevant across contexts than material or orientation, then speakers might disproportionately refer to colour due to its general utility. Although colour and orientation were of objectively equal goal-utility and relevance in our study, speakers may not ignore their prior experience regarding which information is or isn’t generally relevant to their goals. Since listener search is driven by higher-level goals as well as low-level perceptual features (Henderson et al., 2009; Wolfe & Horowitz, 2017), future research should explore how the nature of the referential goal interacts with the listeners’ visual search, going beyond our focus on low-level perceptual features.

To summarise, the present study extends prior work (Jara-Ettinger & Rubio-Fernandez, 2022; Rubio-Fernandez, 2019) and demonstrates that overinformative reference is an efficient communicative strategy that facilitates listener search in complex environments. In this way, our work aligns with recent theoretical advances arguing that communication is optimised for functional utility (Gibson et al., 2019) and speaker-listener co-ordination (Rubio-Fernandez et al., 2025). A further challenge lies in explaining the mechanisms through which speakers learn such strategies and to what extent these strategies are implicit or deliberate. To this question, we anticipate that the prior impressions of perceptual usefulness and goal-relevance play important roles in shaping cross-situational reference strategies.

## ACKNOWLEDGMENTS

The authors thank Paul Garrett and Andrew Perfors for helpful discussions and for their advice on Experiment Two. We also thank the anonymous reviewers for their helpful comments on the original manuscript.

## AUTHOR CONTRIBUTIONS

Merrick Giles: Conceptualization; Formal analysis; Methodology; Visualization; Writing – original draft; Writing – review & editing. Paula Rubio-Fernandez: Conceptualization; Methodology; Writing – review & editing. Francis Mollica: Conceptualization; Formal analysis; Funding acquisition; Methodology; Supervision; Writing – review & editing.

## DATA AVAILABILITY STATEMENT

Processed data, as well as code for experiments and analyses, are available here: https://github.com/merrickgiles/perceptuallyEfficientOverinformativeness.

## Notes

^1^ While the pragmatics literature has also investigated overinformative reference with relative adjectives (Pechmann, 1989; Rubio-Fernandez, 2019; Sedivy, 2003) and numerical modifiers (Wu & Gibson, 2021; Zevakhina et al., 2021), the search efficiency view has focused on absolute adjectives due to differences in semantic computation. Absolute adjectives nicely correspond to perceptual filtering computations; whereas, relative adjectives and numerical modifiers require additional comparative and estimative/enumerative computations.^2^ Some responses were indicative of fatigue effects (e.g., giving up with the experiment and typing *qqq* as the response to all remaining trials). We excluded all trials from such participants.^3^ We conducted a free-response version of the task (*N* = 37, 20 participants assigned to orientation-redundant, 20 to colour-redundant, three exclusions), wherein the colour and orientation stimuli were used, but participants were not provided with response buttons: participants simply typed responses into the next box. This ensures that the pattern of results observed in Experiment Two generalises to free-response paradigms. The results of the Free Response version precisely replicated the results of the main experiment, with strikingly similar proportions of overinformativeness to that of the response selection version. Further details are presented in Supplementary Materials.^4^ The results are qualitatively identical when these trials are retained; see Supplementary Materials.

## Supplementary Material



## References

[bib1] Abbott, J. T., Griffiths, T. L., & Regier, T. (2016). Focal colors across languages are representative members of color categories. Proceedings of the National Academy of Sciences, 113(40), 11178–11183. 10.1073/pnas.1513298113, 27647896 PMC5056040

[bib2] Alexander, R. G., & Zelinsky, G. J. (2011). Visual similarity effects in categorical search. Journal of Vision, 11(8), 9–9. 10.1167/11.8.9, 21757505 PMC8409006

[bib3] Ariel, M. (2014). Accessing noun-phrase antecedents. Routledge. 10.4324/9781315857473

[bib4] Arts, A., Maes, A., Noordman, L., & Jansen, C. (2011). Overspecification facilitates object identification. Journal of Pragmatics, 43(1), 361–374. 10.1016/j.pragma.2010.07.013

[bib5] Battaglia, P. W., Jacobs, R. A., & Aslin, R. N. (2003). Bayesian integration of visual and auditory signals for spatial localization. Journal of the Optical Society of America A, 20(7), 1391–1397. 10.1364/JOSAA.20.001391, 12868643

[bib6] Bejjanki, V. R., Clayards, M., Knill, D. C., & Aslin, R. N. (2011). Cue integration in categorical tasks: Insights from audio-visual speech perception. PLoS One, 6(5), e19812. 10.1371/journal.pone.0019812, 21637344 PMC3102664

[bib7] Brainard, D. H. (2003). Color appearance and color difference specification. In S. K. Shevell (Ed.), The science of color (2nd ed., pp. 191–216). Elsevier. 10.1016/B978-044451251-2/50006-4

[bib8] Bürkner, P.-C. (2017). brms: An R package for Bayesian multilevel models using Stan. Journal of Statistical Software, 80(1), 1–28. 10.18637/jss.v080.i01

[bib9] Clarke, A. D., Elsner, M., & Rohde, H. (2013). Where’s Wally: The influence of visual salience on referring expression generation. Frontiers in Psychology, 4, 329. 10.3389/fpsyg.2013.00329, 23785344 PMC3684789

[bib10] Degen, J., Hawkins, R. D., Graf, C., Kreiss, E., & Goodman, N. D. (2020). When redundancy is useful: A Bayesian approach to “overinformative” referring expressions. Psychological Review, 127(4), 591–621. 10.1037/rev0000186, 32237876

[bib11] Duncan, J., & Humphreys, G. W. (1989). Visual search and stimulus similarity. Psychological Review, 96(3), 433–458. 10.1037/0033-295X.96.3.433, 2756067

[bib12] Egeth, H. E., Virzi, R. A., & Garbart, H. (1984). Searching for conjunctively defined targets. Journal of Experimental Psychology: Human Perception and Performance, 10(1), 32–39. 10.1037/0096-1523.10.1.32, 6242762

[bib13] Engelhardt, P. E., Bailey, K. G., & Ferreira, F. (2006). Do speakers and listeners observe the gricean maxim of quantity? Journal of Memory and Language, 54(4), 554–573. 10.1016/j.jml.2005.12.009

[bib14] Engelhardt, P. E., Demiral, S. B., & Ferreira, F. (2011). Over-specified referring expressions impair comprehension: An ERP study. Brain and Cognition, 77(2), 304–314. 10.1016/j.bandc.2011.07.004, 21840639

[bib15] Ferreira, V. S. (2019). Amechanistic framework for explaining audience design in language production. Annual Review of Psychology, 70, 29–51. 10.1146/annurev-psych-122216-011653, 30231000

[bib16] Ferreira, V. S., & Dell, G. S. (2000). Effect of ambiguity and lexical availability on syntactic and lexical production. Cognitive Psychology, 40(4), 296–340. 10.1006/cogp.1999.0730, 10888342

[bib17] Fukumura, K., & Carminati, M. N. (2022). Overspecification and incremental referential processing: An eye-tracking study. Journal of Experimental Psychology: Learning, Memory, and Cognition, 48(5), 680–701. 10.1037/xlm0001015, 34197167

[bib18] Gatt, A., Krahmer, E., van Deemter, K., & van Gompel, R. P. (2017). Reference production as search: The impact of domain size on the production of distinguishing descriptions. Cognitive Science, 41(S6), 1457–1492. 10.1111/cogs.12375, 27264504

[bib19] Gibson, E., Futrell, R., Piantadosi, S. P., Dautriche, I., Mahowald, K., Bergen, L., & Levy, R. (2019). How efficiency shapes human language. Trends in Cognitive Sciences, 23(5), 389–407. 10.1016/j.tics.2019.02.003, 31006626

[bib20] Gibson, E., Futrell, R., Jara-Ettinger, J., Mahowald, K., Bergen, L., Ratnasingam, S., Gibson, M., Piantadosi, S. T., & Conway, B. R. (2017). Color naming across languages reflects color use. Proceedings of the National Academy of Sciences, 114(40), 10785–10790. 10.1073/pnas.1619666114, 28923921 PMC5635863

[bib21] Giordano, B. L., & McAdams, S. (2006). Material identification of real impact sounds: Effects of size variation in steel, glass, wood, and plexiglass plates. Journal of the Acoustical Society of America, 119(2), 1171–1181. 10.1121/1.2149839, 16521778

[bib22] Grice, H. P. (1975). Logic and conversation. In P. Cole & J. L. Morgan (Eds.), Speech acts: Syntax and semantics (Vol. 3, pp. 41–58). Brill. 10.1163/9789004368811_003

[bib23] Henderson, J. M., Malcolm, G. L., & Schandl, C. (2009). Searching in the dark: Cognitive relevance drives attention in real-world scenes. Psychonomic Bulletin & Review, 16(5), 850–856. 10.3758/PBR.16.5.850, 19815788

[bib24] Itti, L., & Baldi, P. (2009). Bayesian surprise attracts human attention. Vision Research, 49(10), 1295–1306. 10.1016/j.visres.2008.09.007, 18834898 PMC2782645

[bib25] Jara-Ettinger, J., & Rubio-Fernandez, P. (2022). The social basis of referential communication: Speakers construct physical reference based on listeners’ expected visual search. Psychological Review, 129(6), 1394–1413. 10.1037/rev0000345, 34968132

[bib26] Kemp, C., & Regier, T. (2012). Kinship categories across languages reflect general communicative principles. Science, 336(6084), 1049–1054. 10.1126/science.1218811, 22628658

[bib27] Koch, C., & Ullman, S. (1987). Shifts in selective visual attention: Towards the underlying neural circuitry. In L. M. Vaina (Ed.), Matters of intelligence: Conceptual structures in cognitive neuroscience (pp. 115–141). Springer. 10.1007/978-94-009-3833-5_5

[bib28] Koolen, R., Goudbeek, M., & Krahmer, E. (2013). The effect of scene variation on the redundant use of color in definite reference. Cognitive Science, 37(2), 395–411. 10.1111/cogs.12019, 23294102

[bib29] Krauss, R. M., & Glucksberg, S. (1977). Social and nonsocial speech. Scientific American, 236(2), 100–105. 10.1038/scientificamerican0277-100194309

[bib30] Kursat, L., & Degen, J. (2021). Perceptual difficulty differences predict asymmetry in redundant modification with color and material adjectives. Proceedings of the Linguistic Society of America, 6(1), 676–688. 10.3765/plsa.v6i1.5003

[bib31] Leek, M. R. (2001). Adaptive procedures in psychophysical research. Perception & Psychophysics, 63(8), 1279–1292. 10.3758/BF03194543, 11800457

[bib32] Lieder, F., & Griffiths, T. L. (2017). Strategy selection as rational metareasoning. Psychological Review, 124(6), 762–794. 10.1037/rev0000075, 29106268

[bib33] Long, M., Moore, I., Mollica, F., & Rubio-Fernandez, P. (2021). Contrast perception as a visual heuristic in the formulation of referential expressions. Cognition, 217, 104879. 10.1016/j.cognition.2021.104879, 34418775

[bib34] Long, M., & Rubio-Fernandez, P. (2024). Beyond typicality: Lexical category affects the use and processing of color words. In L. K. Samuelson, S. L. Frank, M. Toneva, A. Mackey, & E. Hazeltine (Eds.), Proceedings of the 46th Annual Conference of the Cognitive Science Society (pp. 4925–4930). Cognitive Science Society.

[bib35] Neisser, U. (1964). Visual search. Scientific American, 210(6), 94–103. 10.1038/scientificamerican0664-94, 14183749

[bib36] Paraboni, I., van Deemter, K., & Masthoff, J. (2007). Generating referring expressions: Making referents easy to identify. Computational Linguistics, 33(2), 229–254. 10.1162/coli.2007.33.2.229

[bib37] Pechmann, T. (1989). Incremental speech production and referential overspecification. Linguistics, 27(1), 89–110. 10.1515/ling.1989.27.1.89

[bib38] R Core Team. (2023). R: A language and environment for statistical computing [Computer software manual]. R Foundation for Statistical Computing. https://www.R-project.org/

[bib39] Regier, T., Kay, P., & Khetarpal, N. (2007). Color naming reflects optimal partitions of color space. Proceedings of the National Academy of Sciences, 104(4), 1436–1441. 10.1073/pnas.0610341104, 17229840 PMC1783097

[bib40] Rehrig, G., Cullimore, R. A., Henderson, J. M., & Ferreira, F. (2021). When more is more: Redundant modifiers can facilitate visual search. Cognitive Research: Principles and Implications, 6, 10. 10.1186/s41235-021-00275-4, 33595751 PMC7889780

[bib41] Rosch, E. (1978). Principles of categorization. In E. Rosch & B. B. Lloyd (Eds.), Cognition and categorization (pp. 27–48). Lawrence Erlbaum Associates.

[bib42] Rubio-Fernandez, P. (2016). How redundant are redundant color adjectives? An efficiency-based analysis of color overspecification. Frontiers in Psychology, 7, 153. 10.3389/fpsyg.2016.00153, 26924999 PMC4760116

[bib43] Rubio-Fernandez, P. (2019). Overinformative speakers are cooperative: Revisiting the gricean maxim of quantity. Cognitive Science, 43(11), e12797. 10.1111/cogs.12797, 31742756

[bib44] Rubio-Fernandez, P. (2021). Color discriminability makes overspecification efficient: Theoretical analysis and empirical evidence. Humanities and Social Sciences Communications, 8, 147. 10.1057/s41599-021-00818-6

[bib45] Rubio-Fernandez, P., Berke, M. D., & Jara-Ettinger, J. (2025). Tracking minds in communication. Trends in Cognitive Sciences, 29(3), 269–281. 10.1016/j.tics.2024.11.005, 39694731

[bib46] Sedivy, J. C. (2003). Pragmatic versus form-based accounts of referential contrast: Evidence for effects of informativity expectations. Journal of Psycholinguistic Research, 32(1), 3–23. 10.1023/A:1021928914454, 12647560

[bib47] Sedivy, J. C. (2004). Evaluating explanations for referential context effects: Evidence for Gricean mechanisms in online language interpretation. In J. C. Trueswell & M. K. Tanenhaus (Eds.), Approaches to studying world-situated language use: Bridging the language-as-product and language-as-action traditions (pp. 345–364). MIT Press.

[bib48] Tarenskeen, S., Broersma, M., & Geurts, B. (2015). Overspecification of color, pattern, and size: Salience, absoluteness, and consistency. Frontiers in Psychology, 6, 1703. 10.3389/fpsyg.2015.01703, 26594190 PMC4635207

[bib49] Tatler, B. W., Hayhoe, M. M., Land, M. F., & Ballard, D. H. (2011). Eye guidance in natural vision: Reinterpreting salience. Journal of Vision, 11(5), 5. 10.1167/11.5.5, 21622729 PMC3134223

[bib50] Tourtouri, E. N., Delogu, F., & Crocker, M. W. (2021). Rational redundancy in referring expressions: Evidence from event-related potentials. Cognitive Science, 45(12), e13071. 10.1111/cogs.13071, 34897768

[bib51] Tourtouri, E. N., Delogu, F., Sikos, L., & Crocker, M. W. (2019). Rational over-specification in visually-situated comprehension and production. Journal of Cultural Cognitive Science, 3(2), 175–202. 10.1007/s41809-019-00032-6

[bib52] Traer, J., Cusimano, M., & McDermott, J. H. (2019). A perceptually inspired generative model of rigid-body contact sounds. In Proceedings of the 22nd International Conference on Digital Audio Effects (DAFx-19). https://mcdermottlab.mit.edu/papers/Traer_Cusimano_McDermott_2019_DAFx.pdf

[bib53] van Gompel, R. P., van Deemter, K., Gatt, A., Snoeren, R., & Krahmer, E. J. (2019). Conceptualization in reference production: Probabilistic modeling and experimental testing. Psychological Review, 126(3), 345–373. 10.1037/rev0000138, 30907620

[bib54] Viethen, J., van Vessem, T., Goudbeek, M., & Krahmer, E. (2017). Color in reference production: The role of color similarity and color codability. Cognitive Science, 41(S6), 1493–1514. 10.1111/cogs.12387, 27322921

[bib55] Westerbeek, H., Koolen, R., & Maes, A. (2015). Stored object knowledge and the production of referring expressions: The case of color typicality. Frontiers in Psychology, 6, 935. 10.3389/fpsyg.2015.00935, 26217268 PMC4491598

[bib56] Wickham, H., Averick, M., Bryan, J., Chang, W., McGowan, L. D., François, R., Grolemund, G., Hayes, A., Henry, L., Hester, J., Kuhn, M., Pedersen, T. L., Miller, E., Bache, S. M., Müller, K., Ooms, J., Robinson, D., Seidel, D. P., Spinu, V., … Yutani, H. (2019). Welcome to the tidyverse. Journal of Open Source Software, 4(43), 1686. 10.21105/joss.01686

[bib57] Wnuk, E., Verkerk, A., Levinson, S. C., & Majid, A. (2022). Color technology is not necessary for rich and efficient color language. Cognition, 229, 105223. 10.1016/j.cognition.2022.105223, 36113197

[bib58] Wolfe, J. M. (1998). What can 1 million trials tell us about visual search? Psychological Science, 9(1), 33–39. 10.1111/1467-9280.00006

[bib59] Wolfe, J. M., & Horowitz, T. S. (2004). What attributes guide the deployment of visual attention and how do they do it? Nature Reviews Neuroscience, 5(6), 495–501. 10.1038/nrn1411, 15152199

[bib60] Wolfe, J. M., & Horowitz, T. S. (2017). Five factors that guide attention in visual search. Nature Human Behaviour, 1(3), 0058. 10.1038/s41562-017-0058, 36711068 PMC9879335

[bib61] Wu, S. A., & Gibson, E. (2021). Word order predicts crosslinguistic differences in the production of redundant color and number modifiers. Cognitive Science, 45(1), e12934. 10.1111/cogs.12934, 33452719

[bib62] Zaslavsky, N., Kemp, C., Tishby, N., & Regier, T. (2019). Color naming reflects both perceptual structure and communicative need. Topics in Cognitive Science, 11(1), 207–219. 10.1111/tops.12395, 30457215

[bib63] Zevakhina, N., Pasalskaya, L., & Chinkova, A. (2021). Over-specification of small cardinalities in referential communication. Frontiers in Psychology, 12, 745230. 10.3389/fpsyg.2021.745230, 34912269 PMC8666505

[bib64] Zipf, G. K. (1949). Human behaviour and the principle of least effort. Addison-Wesley.

