# Supplementary material for: Search Efficiency Drives Reference Production Across Modalities, But Colour Is Special
Source: Open Mind (Camb). 2026 Feb 15;10:236–60. doi: 10.1162/OPMI.a.337 (PMC13053021; doi:10.1162/OPMI.a.337)
Supplement: Supplementary file 1 [file opmi-10-236-s001.pdf]

OPEN MIND
Discoveries in Cognitive Science
an open access journal

Citation:

DOI:
http://dx.doi.org/

Supplemental Materials:
http://

Received:
Accepted:
Published:

Competing Interests: The authors have declared that no competing interests exist.

Corresponding Author:

Copyright: © 2026
Massachusetts Institute of Technology
Published under a Creative Commons Attribution 4.0 International
(CC BY 4.0) license

The MIT Press

Supplementary Material for 'Search Efficiency Drives Reference Production Across Modalities, But Colour is Special'

Merrick Giles[1], **Paula Rubio-Fernandez**[2], **Francis Mollica**[1]

[1]University of Melbourne School of Psychological Sciences

[2]Max Planck Institute for Psycholinguistics

DRAFT

SAMPLE SIZE JUSTIFICATION FOR EXPERIMENT ONE

The sample size for experiment one was fixed by budget constraints in the face of large exclusion rates revealed in piloting. We could afford a sample of 120, expecting 40 participants to be excluded. The target sample size of 80 participants is double the usual sample size for director tasks.

PILOT STUDIES AND CHALLENGES FOR EXPERIMENT ONE

We undertook four pilot studies in total for experiment one. For all but the final pilot, participants were overinformative for every trial without exception. We believed that this was due to the perceptual staircases: after having completed two perceptual staircases of colour and material, participants seemed to be under the impression that the task required them to mention both the colour and material for every target item. This is unsurprising given the task structure: after spending 10 minutes completing two tasks where one has to label colours and material-sounds, it is only natural that participants presume they must mention both in the description task.

To overcome this, we implemented a short instruction task where participants took the perspective of their listener (Ralph) just prior to the director task phase. Participants were introduced to their listener: a worker at a baseball bat manufacturing company named Ralph, who would be sorting baseball bats on the basis of their referring description. They were then required to judge whether a given reference was suitable for Ralph (yes/no) across six examples. In three examples, material uniquely demarcated the target referent. In the remaining three examples, colour uniquely demarcated the target referent. In all trials, uniquely demarcating information was given. This task is illustrated in Figure 1.

INTERACTION BETWEEN DISPLAY TYPE AND ATTRIBUTE-REDUNDANT IN EXPERIMENT ONE

While we ran models for each independent predictor (Attribute Redundant and Display Type), the only model with even remotely comparable superiority to that reported in the main text is the same model, but including an interaction between Attribute Redundant and Display Type. Bayes Factor Comparisons revealed that the model including the interaction was marginally inferior to the model without the interaction ($BF = 0.98$ for the model without the interaction). Furthermore, we had no theoretical motivation for including the interaction. The only significant interaction parameter in this model was between Attribute Redundant (Colour vs. Material) and S-Low/R-High Display Type versus Baseline, which was not of primary interest. There was no significant interaction between Attribute Redundant and S-Low/R-High vs S-High/R-Low. Summary statistics for this model are shown

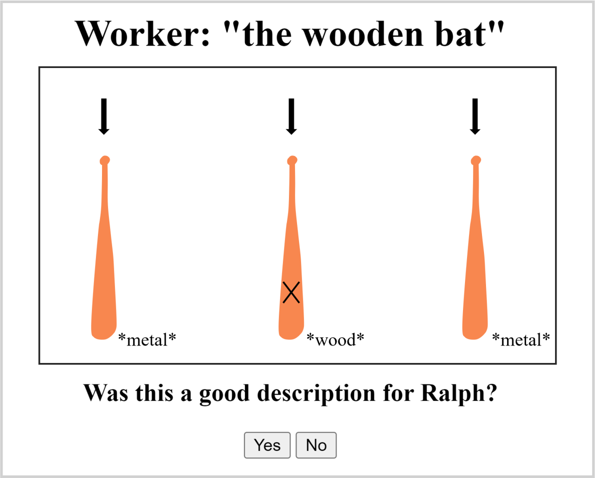

Figure 1. Example Trial of the Interlocutor Task. Initially, the screen was blank. Participants clicked a *play* button at the top of the screen. The bats were presented sequentially, from left to right, with their associated colour and auditory material. The X appeared following all three presentations. In this instance, participants were required to press *yes* and were told that their answer was correct. If they answered incorrectly (*no*), the answer was explained: "the wooden bat provides sufficient information to point out the target."

in Table 1: the reference levels are Colour–Redundant and S-Low/R-High Display Type. Given the lack of evidence for the interactions, in the main text, we only report the model with the two independent and theoretically motivated effects.

Effect	β	SE	95% CI	
			LL	UL
Intercept	-2.47	0.41	-3.31	-1.70
Material Redundant	1.31	0.18	0.96	1.66
S-High/R-Low	1.15	0.18	0.80	1.50
Baseline	0.68	0.18	0.33	1.03
Material : S-High/R-Low	-0.16	0.25	-0.65	0.34
Material : Baseline	0.58	0.26	0.07	1.10

Table 1. Regression Results. SE = Standard Error; 95% CI = Bayesian 95% Credible Intervals.

SAMPLE-SIZE JUSTIFICATION FOR EXPERIMENT TWO

For experiment two, we conducted a simple frequentist power analysis to determine an approximate appropriate sample size. The first experiment we conducted was the free-response paradigm. For this paradigm, we conducted a power analysis using the data collected for experiment one. We achieved this by 1) randomly removing trials from the experiment one data frame, such that the number of trials was equal to that of experiment two (34 critical trials). 2) Setting up a frequentist version of the Bayesian statistical model

reported in the main text. 3) Halving the fixed effect size of Attribute Redundant to quantify the minimal effect of interest (colour vs. material comparison). Finally, we computed power curves for the main effect of Attribute redundant for a range of sample sizes using SimR (**?**). We found that just 20 participants yielded $\sim$ 92% power.

For the response selection paradigm of experiment 2, we conducted a power analysis manually (as SimR comes with the limitation of testing just one effect at a time) with a significance criterion of z = $>$ 2.5 (arbitrary, like all significance tests). For this analysis, we used the data collected from the free-response paradigm in experiment one. Again, we ran a frequentist version of the model and altered the effect size of the main effect (Attribute Redundant). This time we included the effect at three levels: Colour–low frequency (LF), Colour–high frequency (HF), Orientation. The additional colour–low frequency level created by recoding the Orientation Redundant condition of the free-response paradigm. We tested two main possibilities. Possibility 1): Colour–HF would have higher rates of overinformativeness than both Orientation and Colour–LF equally. We tested this by halving the main effect size of Attribute Redundant in the free-response data. The result was $\sim$98% power with just 20 participants. 2): Rates of overinformativeness between Colour–HF and Colour–LF would differ, but to a smaller degree (than Colour–HF vs. Orientation). We achieved this by reducing the main effect size of the Colour–HF and Colour–LF comparison in the model. We found that this comparison reached $\sim$79% power with N=20 by dividing the main effect size by 2.8, and set this as the minimal effect size of interest.

RESULTS FOR THE FREE-RESPONSE PARADIGM OF EXPERIMENT TWO

Before both free-response and the response selection paradigms of experiment two, we conducted pilot studies with $N = 5$. Finding that there were no experimental issues, we proceeded with data collection. We conducted a free-response version of the task (N = 40: 20 participants assigned to orientation-redundant, 20 to colour-redundant), wherein the colour and orientation stimuli were used, but participants were not provided with response buttons: participants simply typed responses into the next box. (Figure 2) [1] That is, the free-response paradigm follows the response selection experiment reported in the main text, with just one modification: instead of pressing buttons to construct their expressions, participants typed freely. This redundancy now has benefits: we can confirm that the pattern of results observed in Experiment Two does indeed generalise to more natural free-response paradigms.

From our initial sample of 40 participants, we removed a total of three participants; two were removed for failing to establish reference on $>$ 50% of critical trials, and one was removed for violating task instructions (using spatial location such as ”the one on the right”) on $>$ 50% of trials. After these exclusions, we removed individual trials. We restricted analysis to trials with established reference:[2] this resulted in the removal of 15 trials (.01%). We also removed 38 trials whereby participants used spatial information (.03%).

[1] We initially conducted this Free-response version before the button experiment as a standalone (see preregistration: osf.io/wtfqk). As Experiment Two (with buttons) makes the experiment redundant, the experiment is only informative insofar as it shows the results extend to free-response paradigms.

[2] Due to server error, we lost data on the ground truth for shape data: establishing reference was therefore defined as any mention of shape. This was likely of minimal influence, as there were no participants excluded in the response–selection task for this reason

The remaining data for critical (non-filler) trials consisted of 37 participants, with a total of 1205 trials. Data was visualised and analysed using R **?** in the Tidyverse **?**. As in the main text, we employed Bayesian Logistic Regressions (intercept and main effects) using brms **?** with $\mathcal{N}(0, 2)$ priors over regression coefficients, chosen using prior-predictive checks. The predictor of Attribute-redundant (orientation, colour) was coded using sum contrasts.

The results of the Free Response condition were precisely in line with the results of the main experiment (with remarkably similar proportions), with overinformativeness on colour-redundant trials far exceeding that of orientation-redundant trials, with highly similar proportions to the response selection experiment (Figure 3). Statistical results are presented in Table 2.

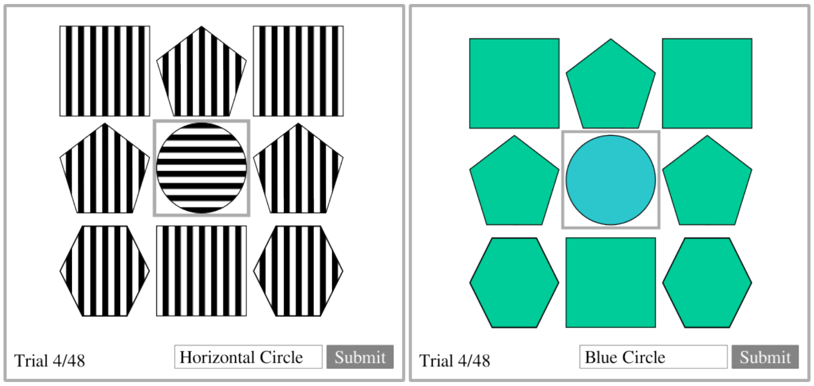

Figure 2. Illustration of the Free Response Paradigm. Text boxes show example responses; in the experiment, the text boxes were empty until participants provided responses.

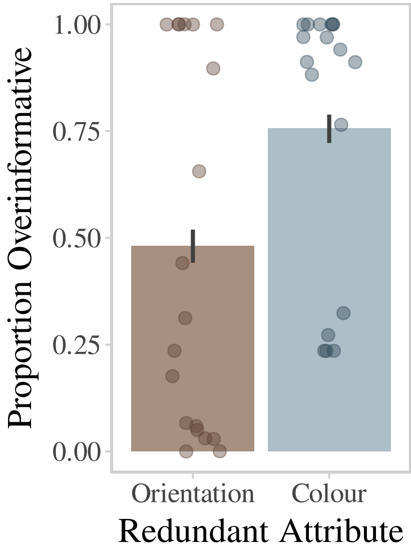

Figure 3. Dots represent individual participant means. Lines represent bootstrapped 95% confidence intervals.

Effect	β	SE	95% CI	
			LL	UL
Intercept	1.13	0.10	0.94	1.32
Orientation	-1.21	0.12	-1.45	-0.97

Table 2. Regression Results. SE = Standard Error; 95% CI = Bayesian 95% Credible Intervals.

ROBUSTNESS ANALYSIS FOR EXPERIMENT TWO: NO TRIAL EXCLUSIONS

In Experiment Two, we removed 44 trials due to participants labelling nouns inaccurately. A qualitative check revealed that this was commonly due to participants labelling hexagons as pentagons or vice versa. Following prior research, we removed inaccurate trials. To ensure our results were not due to these inclusions (for whatever reason), we analysed the results while retaining all trials (no removals). This resulted in an analysis using 2038 trials (as opposed to the 1994 in the main text analysis). The results were qualitatively identical to the analysis with exclusions (Table 3).

Effect	β	SE	95% CI	
			LL	UL
Intercept	0.97	0.08	0.81	1.14
LF Colour Terms Redundant	-0.19	0.12	-0.43	0.04
Orientation Redundant	-0.97	0.12	-1.20	-0.74

Table 3. Regression Results. Reference Level: High Frequency Colour Terms; SE = Standard Error; 95% CI = Bayesian 95% Credible Intervals.
